# A treatment planning study of proton arc therapy for para-aortic lymph node tumors: dosimetric evaluation of conventional proton therapy, proton arc therapy, and intensity modulated radiotherapy

**DOI:** 10.1186/s13014-016-0717-4

**Published:** 2016-10-21

**Authors:** Jeong-Eun Rah, Gwe-Ya Kim, Do Hoon Oh, Tae Hyun Kim, Jong Won Kim, Dae Yong Kim, Sung Yong Park, Dongho Shin

**Affiliations:** 1 Department of Radiation Oncology, Myongji Hospital, Seonam University College of Medicine, Goyang, Korea; 2 Department of Radiation Medicine and Applied Sciences, University of California, San Diego, CA USA; 3 Proton Therapy Center, National Cancer Center, Goyang, Korea; 4 Department of Radiation Oncology, Konyang Hospital, Daejeon, Korea; 5 McLaren Proton Therapy Center, Karmanos Cancer Institute, Flint, MI USA

**Keywords:** Proton arc therapy (PAT), Proton beam therapy (RBT), Intensity modulated radiation therapy (IMRT), Organ equivalent doses (OED), Normal tissue complication probability (NTCP)

## Abstract

**Background:**

The purpose of this study is to evaluate the dosimetric benefits of a proton arc technique for treating tumors of the para-aortic lymph nodes (PALN).

**Method:**

In nine patients, a proton arc therapy (PAT) technique was compared with intensity modulated radiation therapy (IMRT) and proton beam therapy (PBT) techniques with respect to the planning target volume (PTV) and organs at risk (OAR). PTV coverage, conformity index (CI), homogeneity index (HI) and OAR doses were compared. Organ-specific radiation induced cancer risks were estimated by applying organ equivalent dose (OED) and normal tissue complication probability (NTCP).

**Results:**

The PAT techniques showed better PTV coverage than IMRT and PBT plans. The CI obtained with PAT was 1.19 ± 0.02, which was significantly better than that for the IMRT techniques. The HI was lowest for the PAT plan and highest for IMRT. The dose to the OARs was always below the acceptable limits and comparable for all three techniques. OED results calculated based on a plateau dose–response model showed that the risk of secondary cancers in organs was much higher when IMRT or PBT were employed than when PAT was used. NTCPs of PAT to the stomach (0.29 %), small bowel (0.69 %) and liver (0.38 %) were substantially lower than those of IMRT and PBT.

**Conclusion:**

This study demonstrates that there is a potential role for PAT as a commercialized instrument in the future to proton therapy.

## Introduction

New technologies in the delivery of radiation therapy have included the use of intensity-modulated radiation therapy (IMRT) or volumetric modulated arc therapy (VMAT) with linear accelerators, as well as the development of proton beam therapy (PBT), which has increased the ability to maximize the dose to the tumor while sparing normal structures. The VMAT approach has a number of potential advantages compared to IMRT, such as significantly reducing the treatment time and the number of MUs, as well as improving normal tissue sparing while keeping adequate coverage. Also, proton beams, unlike X-ray beams, have a low entrance dose, followed by a region of uniform high dose (the spread out Bragg peak) at the target, and then a steep fall-off to zero dose. These characteristics minimize the dose delivered to normal tissues while maximizing the dose delivered to the tumor. Better or comparable dose conformity with decreased low dose volume can be achieved with proton beams than with advanced photon techniques [1–3]. This is because of the advantageous physical properties of protons, including a near zero exit or distal dose just beyond the target volume, resulting in reduced proton doses to normal tissue, with better conformation of the dose to the target volume. These unique dose characteristics of protons may reduce the risk of acute as well as late side effects [4].

In recent years, several studies on treatment planning or dosimetric validation with those obtained by proton arc therapy (PAT) can be found in the literature [5, 6]. Rechner et al. [5] assessed the predicted risk of a second cancer following proton arc therapy and VMAT technique for prostate cancer. This study used proton arc plans with 16 equally spaced, static, passively-scattered proton beams. They reported that the predicted risk of cancer for an out-of-field organ such as the bladder or rectum following proton arc therapy is either less than or approximately equal to the risk with VMAT. Seco et al. [6] compared a proton arc technique using passively scattered beams and IMPT for early-stage non-small cell lung cancer. Their study used 8–14 beams with a maximum arc of 150°. They observed that passive-arc therapy produced comparable tumor conformity to VMAT and significantly reduced the low dose to the lungs.

Although the proton arc technique has been reported in the literature, extensive studies on treatment planning or dosimetric validation of PAT plans have not yet been conducted. Therefore, the purpose of the present study was to compare dosimetric properties of PAT, PBT, and IMRT techniques for tumors of the para-aortic lymph nodes (PALN). We also compared the dose distribution, organ equivalent doses (OED) and normal tissue complication probability (NTCP) resulting from IMRT, PBT, and PAT techniques in the nine patients based on analysis of dose-volume histograms (DVHs).

## Methods and materials

### Patient data and planning techniques

We randomly selected nine patients who were to be treated with IMRT or PBT for PALN tumors at the National Cancer Center (NCC) in Korea. The proton system consists of a 230 MeV proton cyclotron, two gantries with rotating beamlines and one gantry with a stationary horizontal beamline. The two gantries with rotating beamlines utilize passive scattering and uniform scanning delivery techniques. The horizontal beam is usually used to treat tumors of the eyes and prostate cases. The maximum extracted beam current of the cyclotron is 300 nA at a 106 Hz radio frequency. Minimum and maximum ranges of the proton beam in water are 5 and 28 cm, respectively, with 0.1 cm accuracy. When data from all nine patients were analyzed, proton therapy was simulated to prescribed dose with the beam range of 7.59 to 17.28 mm and a modulation width of 5.48 to 11.78 mm.

For all patients, plans were designed on a CT scan (RT 16 PRO CT Simulator, General Electric Medical Systems, Waukesha, WI) acquired with 2.5 mm slice thickness extending the scan from the 11th thoracic vertebral level to include the proximal third of the femur’s diathesis. Patients were simulated in the supine position without a custom immobilization device, and these images were then imported into Eclipse (version 10.0.28, Varian Medical Systems, Palo Alto, CA) treatment planning system (TPS). Gross tumor volumes (GTV) were determined based on CT. Clinical target volumes (CTV) were considered to be identical to the GTV. Planning target volumes (PTV) were delineated as the CTV plus a 10 mm margin in all directions. The organs at risk (OAR) considered were: stomach, small bowel, kidney, liver, and spinal cord. A total dose of 60 Gy was prescribed to the PTV, in 30 fractions of 2Gy per fraction for all patients. Proton doses were corrected with the accepted relative biologic effectiveness (RBE) value of 1.1 [7]. The Eclipse pencil beam algorithm was used to calculated dose. All treatment planning was calculated by using a tissue heterogeneities method and the grid size of calculation was 2.5 mm.

The clinical IMRT plans were generated with 5–7 coplanar fields of a 6 MV photon beam. All IMRT optimizations were done by interactively adapting the objectives and their priorities. A renal tolerance dose of 15 Gy (5 % risk at 5 years) and 20 Gy (50 % risk at 5 years) was assumed [8]. The hepatic tolerance dose (50 % risk at 5 years) was set at 30 Gy for the whole organ [9]. For the spinal cord, a maximum dose objective of 45 Gy was accepted. The maximum dose objective for the stomach was 45 Gy for the entire organ.

The PBT and PAT techniques were generated using a planning system developed specifically for the planning of proton treatments using the so-called passive scattering technique. In systems that use beam scattering to spread the beam, the small beam coming into the nozzle is scattered to a large area, and the scatters are specially designed so that the beam has a uniform penetration and uniform intensity across the scattered area specified for clinical use [10]. At the same time, the energy of the beam is modulated to spread out the location of the Bragg peaks over the target volume in depth. The system is usually configured to produce a homogeneous dose distribution with the same penetration across the beam. Passive scattering proton beams utilize physical patient field-specific hardware, such as aperture and range compensator to obtain better conformation of the dose to the target volume. The patient aperture is a brass beam stop with a hole shaped to the outer projection of the target in the beam’s eye view. The range compensator is a plastic block and may be designed for diverse clinical goals: guaranteeing target coverage in the face of alignment error, patient and internal organ motion, and assuring the neighboring critical structures are spared [3]. For the PBT treatment planning, two-field or three-field beams were used and the angles of 0 or 180 and 270 or 90 with ± 10° correction to avoid critical structures, especially the small bowel. Since PAT has not been implemented to the current clinical application, the purpose of treatment planning was to design, for each treatment field, the aperture and range compensator, and to specify the SOBP range, modulation and dose from the virtual instrument of PAT. Eclipse TPS was used to split the arcs into beam ports every 5°, resulting in 48 beam ports for 240° arc. At NCC, proton beam energy uncertainty used a systematic range of uncertainty of ± 0.6–1.0 mm; however, because of the physical introduction of patient field-specific hardware in the beam line, the reproducibility of the range plus an additional ±1.0 mm for both PBT and PAT.

### Evaluation parameters

For each plan, DVHs were calculated for the PTV and OARs. The coverage of PTV was calculated as the ratio of target volume covered by 98 % isodose line divided by the volume of PTV. Other criteria for PTV were the mean dose (D_mean_), D_98%,_ D_2%_, (the percentage of prescribed dose delivered to 98 and 2 % of the volume), conformity index (CI) and homogeneity index (HI). The CI is the ratio of target volume covered by 98 % isodose line divided by the total volume covered by that isodose line. A conformity index equal to 1 corresponds to ideal conformation. A conformity index greater than 1 indicates that the irradiated volume is greater than the target volume and includes healthy tissues. If the conformity index is less than 1, the target volume is only partially irradiated [11]. According to RTOG guidelines, ranges of conformity index values have been defined to determine the quality of conformation, because a value of 1 is rarely obtained. If the conformity index is between 1 and 2, treatment is considered to comply with the treatment plan [12]. The HI describes the uniformity of dose within a treated target volume, and is directly calculated by the steepness of the target DVH about the prescription dose, i.e. the difference in dose received by 2 and 98 % of the PTV D_2_ − D_98_. According to this definition, D_2_ and D_98_ are considered the maximum and minimum doses, respectively. If the HI index is ≤2, treatment is considered to comply with the RTOG protocol. A lower HI is indicative of a more homogeneous target dose. To quantify the dose distribution of the OARs, V_30%_, V_60%_, V_90%_ (the percentage of the volumes receiving at least 30 %, 60 %, and 90 % of the prescribed dose), and D_mean_ were evaluated.

DVHs were also analyzed in terms of OEDs and NTCPs for the OARs. When analyzing high or medium dose levels organs typically receive inhomogeneous dose distributions. To consider such effect, one might use the concept of OED, in which any dose distribution corresponds to the same OED if it causes the same radiation-induced cancer risks. A risk factor is then applied to the OED [13, 14]. Parameters for OED are the organ-specific cancer incidence rate at low doses, which can be taken from the data of the atomic bomb survivors, and cell sterilization at higher doses [15]. If the true dose–response curves for radiation-induced cancer were known for each organ and tissue, an OED estimate would be a perfect parameter to quantify second cancers. However, because the underlying dose–response function is not known, several models have been used. One can thus assume three dose–response relationships, linear, bell-shaped, and plateau-shaped using free model parameter *a*_*organ*_ and *δ*_*organ*_ [16] as follows:

- linear model $$ OED=\frac{1}{V}{\displaystyle {\sum}_i{V}_i{D}_i,} $$
- bell-shaped model $OED=\frac{1}{V}{\displaystyle {\sum}_i{V}_i{D}_i \exp \left(-{a}_{organ}{D}_i\right)}$ and
- plateau model $$ OED=\frac{1}{V}{\displaystyle {\sum}_i{V}_i\left(\frac{1- exp\left(-{\delta}_{organ}{D}_i\right)}{\delta_{organ}}\right),} $$

where *V* is the total organ volume and the sum is taken over all volume element *V*_*i*_ with homogeneous dose *D*_*i*_. Since for small doses the dose–response relationship for cancer induction is with good precision a linear function of dose, OED is simply average orang dose. However, for high doses there is currently much debate concerning the shape of the dose–response curve for radiation-induced cancer [16]. In this study, all three models were presented; however, to estimate secondary cancer risk for the three techniques, we used a plateau dose–response model, which is located approximately in the middle of the two extreme models.

The NTCP was computed using the Lyman Kutcher Burman (LKB) model to ascertain the expected incidence of complications [17]. It consists of a probit equation as the relation between a dose of uniform radiation and the probability of effect. This is combined with an equivalent uniform dose (EUD) DVH reduction method [18]. NTCP can then be calculated as

$$ NTCP=\frac{1}{\sqrt{2\pi }}{\int}_{-\infty}^t{e}^{-\frac{x^2}{2}dx}\;\mathrm{with}\;t=\frac{EUD-{D}_{50}}{m\times {D}_{50}},\;EUD={\left({\displaystyle {\sum}_i^N{v}_i\;{D}_i^{\frac{1}{n}}}\right)}^n $$

*D*_*i*_ and *v*_*i*_ are N pairs of dose level and corresponding subvolume (relative to the total organ volume) of the differential DVH. Three model parameters (*D*_*50*_, *m*, and *n*) can be found in these expressions. *D*_*50*_ is the uniform dose corresponding to 50 % complication probability and *m* is the slope of the dose–response curve at that position. The volume effect is accounted for by parameter *n*. A special case of the LKB model is the mean dose model where *n* is fixed at a value of 1. In order to calculate OED and NTCP, DVH data from each plan was analyzed by using the tool developed with home-made software [19].

The PAT plan was each compared with the IMRT and PBT plans with regard to DVHs and doses to OARs. The paired *t* test was used for all statistical comparisons, with a *p* values of ≤0.05 considered significant; *p* values of < 0.001 were truncated and noted as *p* < 0.001.

## Results

**Fig. 1:**
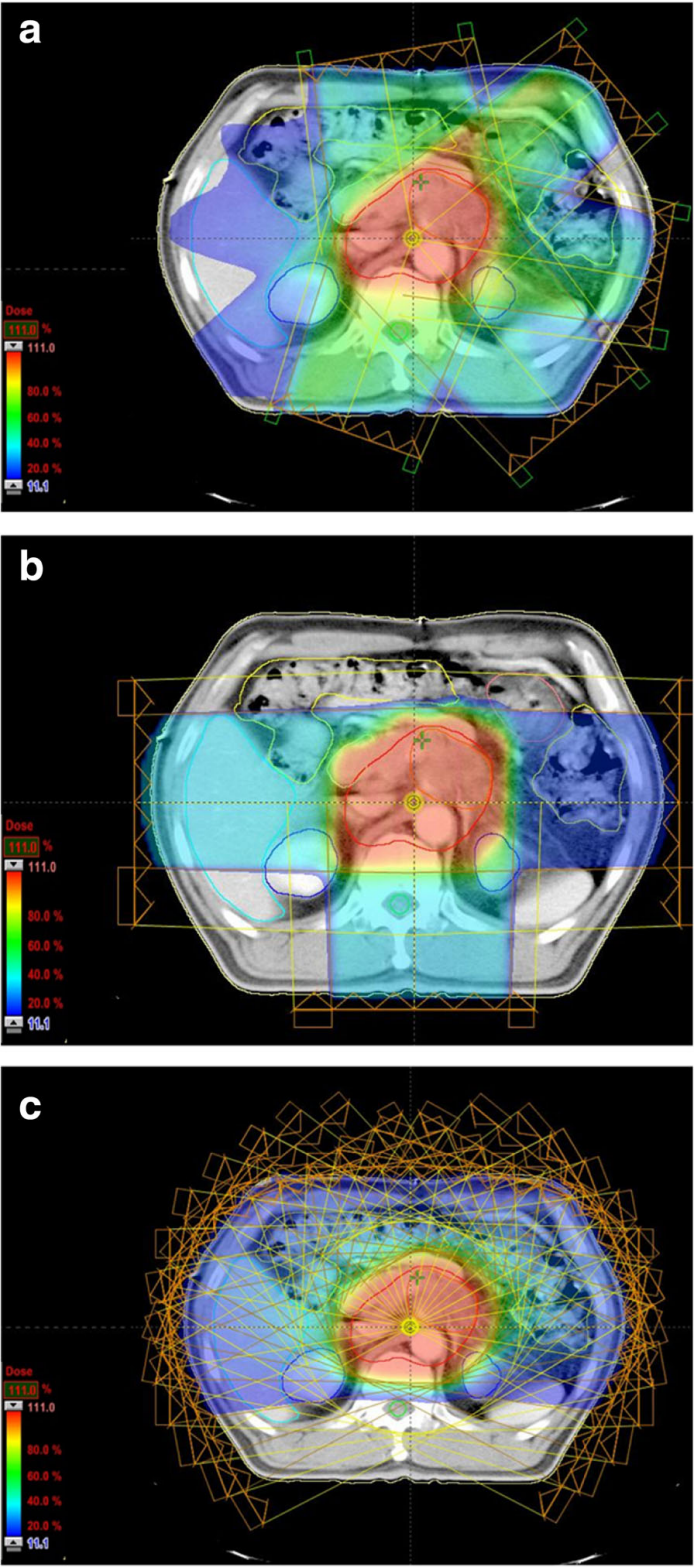
Dose distribution of (**a**) IMRT, (**b**) PBT, and (**c**) PAT technique in axial plane. Color wash banding is restricted to relative dose range of 11–110 %

**Fig. 2:**
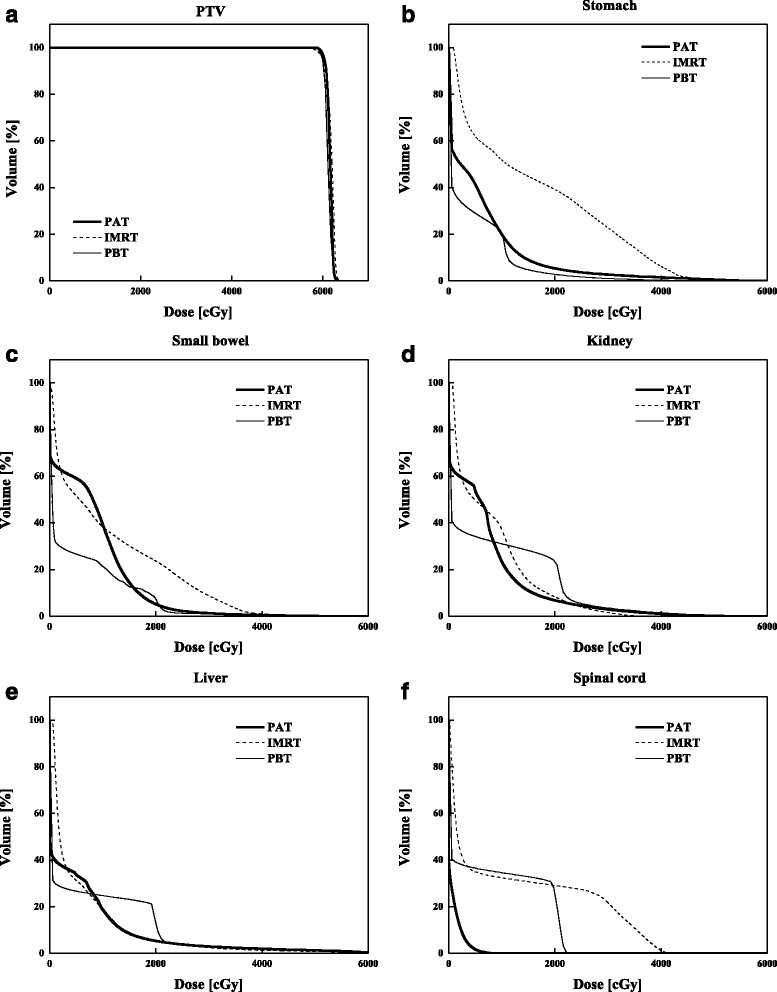
Dose-volume histograms for (**a**) planning target volume (PTV), (**b**) stomach, (**c**) small bowel, (**d**) kidney, (**e**) liver, and (**f**) spinal cord, comparing IMRT, PBT, and PAT techniques

Figure 1a, b and c shows the IMRT, PBT and PAT calculated dose distributions of one patient with PALN tumor, respectively, and then the DVH curves for PTV and OARs of each technique are shown in Fig. 2. Comparing the dose distributions, the PAT plan shows a good conformation of the target volume, with a maximum dose in the target of 106 %. Detailed dosimetric comparisons of the PTV and various OARs for the three techniques, based on DVHs analysis is shown in Table 1. Data are presented as averages over the nine investigated patients and errors indicate inter-patient variability at the 1 standard deviation level. PAT plans had excellent coverage of the PTV with ≥99.6 % of the PTV receiving ≥98 % of the prescribed dose. The CI obtained with PAT was 1.19 ± 0.02, which was significantly better than that for IMRT techniques (*p* < 0.001). The HI was also lowest for PAT plan and highest for IMRT (*p* = 0.003). D_mean_ of the PTV was equal for all three techniques (IMRT vs. PAT (*p* = 1.000), PBT vs. PAT (*p* = 0.371)), whereas D_2%_ was smallest for PAT and highest for IMRT plan (*p* = 0.002). Doses to the OAR were below the acceptable limits and comparable for all three techniques. The PBT technique reduced the D_mean_ more than IMRT techniques in the OAR (*p* = 0.045), which was similar to those of PAT (*p* = 0.692). However, the average OARs volume percentage receiving 30 % of the prescribed dose (V_30%_) was generally lower for PAT compared to PBT for considered OARs (*p* = 0.393).

**Table 1:** Plan comparison between IMRT, PBT and PAT. (average of nine patients)

	IMRT	PBT	PAT	P*
PTV				
Coverage (%)	98.5 ± 0.02	99.0 ± 0.01	99.6 ± 0.01	IMRT vs. PAT (1.000), PBT vs. PAT (0.145)
D_2%_ (Gy)	63.2 ± 0.04	62.8 ± 0.04	62.4 ± 0.02	IMRT vs. PAT (0.002), PBT vs. PAT (1.000)
D_98%_ (Gy)	59.1 ± 0.05	59.4 ± 0.01	59.8 ± 0.02	IMRT vs. PAT (1.000), PBT vs. PAT (1.000)
D_mean_ (Gy)	61.9 ± 0.02	61.4 ± 0.03	61.6 ± 0.03	IMRT vs. PAT (1.000), PBT vs. PAT (0.371)
Conformity index (CI)	1.47 ± 0.03	1.23 ± 0.03	1.19 ± 0.02	IMRT vs. PAT (<0.001), PBT vs. PAT (1.000)
Homogeneity index (HI)	6.83 ± 0.02	5.67 ± 0.01	4.33 ± 0.02	IMRT vs. PAT (0.003), PBT vs. PAT (<0.001)
Stomach				
V_30%_ (%)	41.5 ± 4.1	26.4 ± 14.3	23.7 ± 8.1	IMRT vs. PAT (<0.001), PBT vs. PAT (<0.001)
V_60%_ (%)	12.5 ± 1.5	14.8 ± 5.4	11.3 ± 5.6	IMRT vs. PAT (<0.001), PBT vs. PAT (0.018)
V_90%_ (%)	0.09 ± 0.4	4.25 ± 2.1	3.15 ± 0.9	IMRT vs. PAT (<0.001), PBT vs. PAT (0.002)
D_mean_ (Gy)	16.2 ± 1.6	9.84 ± 3.2	10.1 ± 8.4	IMRT vs. PAT (<0.001), PBT vs. PAT (0.518)
Small bowel				
V_30%_ (%)	26.2 ± 7.5	17.5 ± 10.2	12.7 ± 4.2	IMRT vs. PAT (<0.001), PBT vs. PAT (<0.001)
V_60%_ (%)	2.50 ± 4.2	0.63 ± 4.3	0.73 ± 1.1	IMRT vs. PAT (<0.001), PBT vs. PAT (0.327)
V_90%_ (%)	0.20 ± 1.3	0.11 ± 1.2	0.02 ± 0.4	IMRT vs. PAT (0.402), PBT vs. PAT (0.039)
D_mean_ (Gy)	18.7 ± 1.1	14.9 ± 2.9	15.8 ± 1.9	IMRT vs. PAT (0.365), PBT vs. PAT (0.521)
Kidney				
V_30%_ (%)	12.0 ± 6.3	0.96 ± 2.4	1.10 ± 3.5	IMRT vs. PAT (<0.001), PBT vs. PAT (0.960)
V_60%_ (%)	1.46 ± 3.9	0	0	IMRT vs. PAT (0.053), PBT vs. PAT (0.321)
V_90%_ (%)	0.36 ± 1.4	0	0	IMRT vs. PAT (1.000), PBT vs. PAT (1.000)
D_mean_ (Gy)	6.61 ± 2.9	1.10 ± 3.3	2.41 ± 2.6	IMRT vs. PAT (1.000), PBT vs. PAT (0.412)
Liver				
V_30%_ (%)	10.5 ± 0.9	8.91 ± 6.7	8.02 ± 4.5	IMRT vs. PAT (<0.001), PBT vs. PAT (1.000)
V_60%_ (%)	0.16 ± 0.7	2.16 ± 2.6	1.62 ± 1.7	IMRT vs. PAT (<0.001), PBT vs. PAT (0.852)
V_90%_ (%)	0.02 ± 0.4	0.02 ± 1.3	0.05 ± 1.5	IMRT vs. PAT (0.532), PBT vs. PAT (1.000)
D_mean_ (Gy)	10.6 ± 0.4	7.38 ± 1.9	7.88 ± 1.2	IMRT vs. PAT (1.000), PBT vs. PAT (1.000)
Spinal cord				
V_30 %_ (%)	29.7 ± 7.7	21.5 ± 9.4	0	IMRT vs. PAT (<0.001), PBT vs. PAT (<0.001)
V_60 %_ (%)	8.68 ± 8.2	0	0	IMRT vs. PAT (<0.001), PBT vs. PAT (1.000)
V_90 %_ (%)	0	0	0	IMRT vs. PAT (1.000), PBT vs. PAT (1.000)
D_mean_ (Gy)	15.0 ± 4.2	6.21 ± 6.5	0.92 ± 0.7	IMRT vs. PAT (<0.001), PBT vs. PAT (1.000)

Abbreviations *D*_*x*%_dose received by the x% of the volume, *V*_*x*%_ volume receiving at least x% of the prescribed dose. Average parameters ± standard deviation (k = 1) are displayed

*p values of ≤0.05 considered significant; *p* values of <0.001 were truncated and noted as *p* < 0.001

**Fig. 3:**
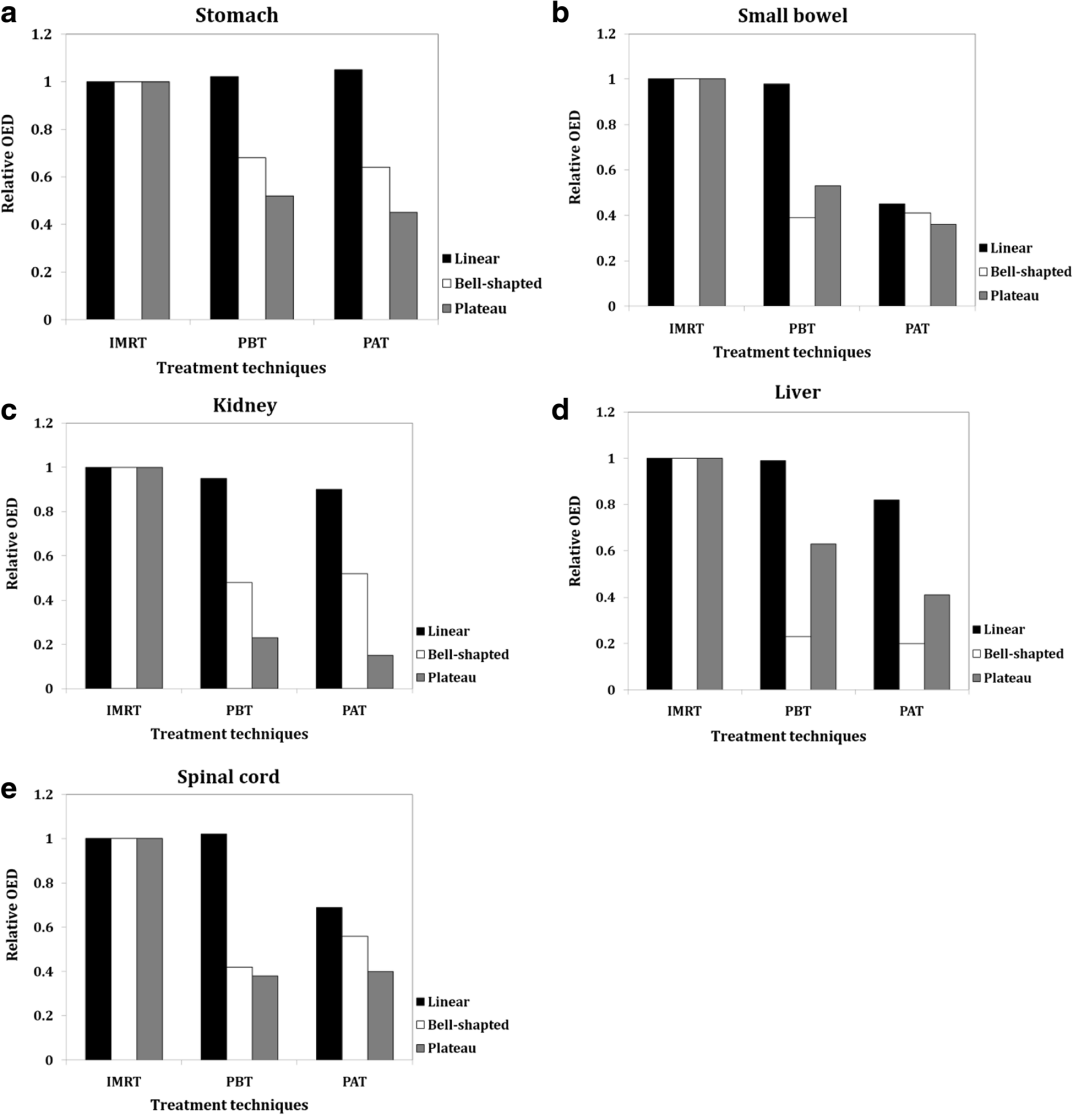
Relative organ equivalent dose (OED) of the (**a**) stomach, (**b**) small bowel, (**c**) kidney, (**d**) liver, and (**e**) spinal cord, using IMRT, PBT and PAT technique to treat nine patients, normalized relative to OEDs of IMRT, using three calculation models

Figure 3 shows the relative OEDs calculated based on a linear, bell-shaped and plateau dose–response model for PALN tumor treatment. The calculated OEDs for the stomach, small bowel, kidney, liver, and spinal cord were normalized to the OEDs of IMRT treatment to determine differences that might be seen when using PBT or PAT. In our results, the risk for IMRT is enhanced in the OARs when applying a bell-shaped and plateau dose–response relationship; however, it is not enhanced for a linear model. For OED calculated with the plateau model, the secondary cancer risks to the OARs were generally lower using PAT than using PBT technique. The OEDs from the IMRT technique were nearly three times higher than those from PAT technique.

**Fig. 4:**
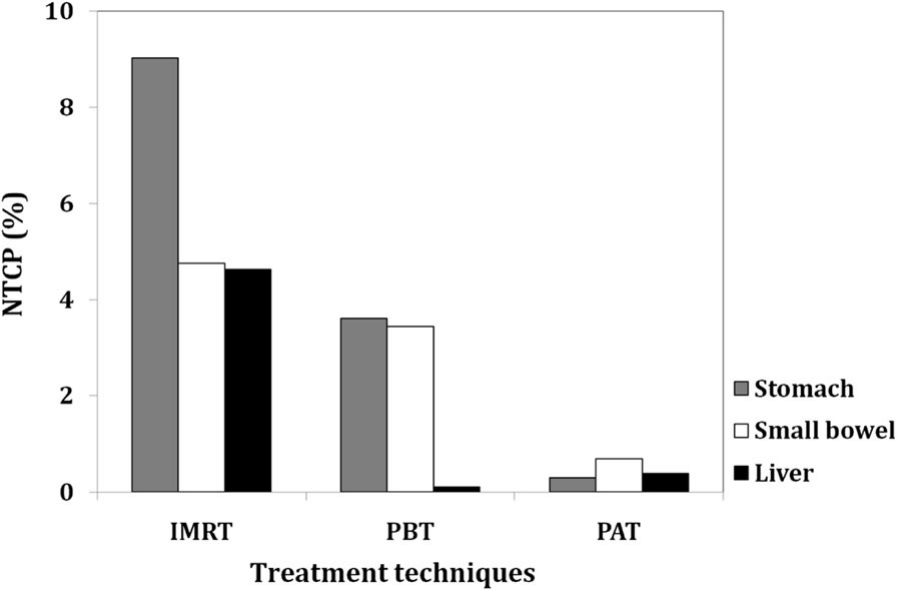
Relative normal complication probability (NTCP) of the stomach, small bowel, and liver using IMRT, PBT technique and PAT to treat nine patients

The calculated values of NTCP for the OARs are shown in Fig. 4 for the three techniques. The estimated NTCP for the kidney as well as for the spinal cord was negligible for any of the treatment techniques and is not shown. In all the investigated OARs, the IMRT technique showed the highest NTCPs. PAT plans reduced NTCP substantially compared to the conventional PBT technique, and our NTCP data for the liver were very similar in both proton plans.

## Discussion

Our findings indicate that PAT resulted in improvements in most dosimetric parameters for PALN tumor patients compared with the IMRT and PBT techniques. The recommendations of our radiation therapy oncology group for dose homogeneity are as follows: no more than 20 % of any PTV will receive > 110 % of the prescribed dose and the prescription dose is the isodose that encompasses at least 95 % of the PTV [20]. This suggests that, for an acceptable plan, 95 and 110 % may be safely taken as the minimum (D_98%_ PTV) and maximum (D_2%_ PTV) doses, respectively. When compared with conventional proton beam plans, PAT technique showed excellent dosimetric results for target coverage, conformity, and homogeneity. PAT also decreased the average OARs volume percentage receiving 30 % of the prescribed dose (V_30%_) compared with PBT and IMRT. Theoretically, PAT technique has the potential to achieve these normal tissue constraints while permitting dose escalation to the grossly enlarged metastatic lymph nodes in the para-aortic area, which was similar to those of other new normal tissue-sparing techniques, such as stereotactic body radiation therapy (SBRT). This study provides further evidence that PAT reduces the risk of radiation side effects because of its superior dosimetric properties.

For the three techniques, we calculated the OED of the OARs. Since OED is directly proportional to the risk of radiation induced cancer different treatment modalities can be compared with respect to their carcinogenic potential. One of the main advantages of conventional proton beam may be the reduced risk of radiation induced secondary malignancy. Secondary cancer risk after prostate and head-and-neck radiotherapy shows the already observed behavior of a modest increase for IMRT than those of proton therapy [21]. Mu et al. [22] estimated the risk of radiation-induced cancer after spinal irradiation for childhood medulloblastoma, and found that the life time risk of secondary cancer was approximately eightfold less when intensity modulated proton therapy (IMPT) was used, compared with IMRT. Recently, another study has shown that the estimated secondary cancer risk associated with proton beams in the treatment of pediatric tumor ranged from 0.6 to 1.5 [23]. In our results, the average OEDs calculated according to a plateau model, after PAT was found in practice to be 0.57, which shows that the secondary malignancy risk associated with PAT is close to that of PBT. This suggests that the risk of secondary cancer after PAT is roughly the same as that after PBT. These estimations are relatively consistent with respect to previously reported data; our results include a certain level of uncertainty as the real incidence of secondary cancers after radiation treatment is not definitively known. Although risk estimations will vary with the model chosen, PBT or PAT seem to minimize secondary cancer risk if one assumes that the lower the radiation dose to OARs, the less the incidence of secondary cancers.

NTCP represents the complication probability of normal organs exposed to radiation. In a recent treatment planning study, the arc technique, such as VMAT distributes lower dose over a larger volume of normal tissue than a static IMRT plan [24]. They showed that VMAT allows one to keep the same PTV coverage with an improved homogeneity and better conformality and, at the same time, presented a major reduction of irradiation of bladder, rectum and small bowel over the entire medium to high dose levels with highly statistically significant differences. If expected toxicity at 50 Gy can be low, the NTCP estimates showed that VMAT, assuming the same quality of plans, could allow some dose escalation process keeping the relative complication risk much smaller than IMRT. In our study, the NTCPs of PAT technique are 0.29 % (stomach), 0.69 % (small bowel), and 0.38 % (liver). It would be possible with a similar sophisticated approach in PAT to further reduce the dose to the PALN tumor. The NTCP estimates showed that PAT, assuming the same quality of plans, could allow some dose escalation process while keeping the relative complication risk low. Altogether, the findings suggest that PAT should be considered as a treatment option for PALN patients and likely for a wide group of abdomen indications (including pancreas, kidney, and liver treatments).

The present study has a few important issues. One issue is that we used approximately 48 beams of one ration for the PAT plans; this means it is needs to be designed for each treatment field, the range modulator and compensator. It would be impractical to deliver these plans in patients using currently-available passive systems. However, it is possible that the alternative method will be found to reduce the number of arc beams for conventional passive scattering beams. This approach was similar to that of Seco et al. [6], who used 8–14 passive-arc beams. Strictly speaking of course, field-specific range compensators are not required for the pencil beam scanning, as the pull-back Bragg peaks can be achieved by an energy adjustment, but they may be useful in terms of the resolution [10, 25]. The spatial resolution of a compensator, typically 3 mm, compares favorably to the size of pencil beams available for IMPT treatments with FWHM of 10 mm and higher. These pencil beam widths are primarily controlled by the beam line magnets but are adversely affected by the traversal of the proton beam through materials to reach the patient. The high lateral resolution of the range compensator, combined with a depth resolution of better than 1 mm, may allow for better conformality to the distal target surface than can be achieved with the energy modulation alone. In IMPT, the number of required irradiation layers may be reduced with the use of compensator. Recently, a variety of devices have become available for scattering and range compensation. An innovative method to make patient-specific hardware, such as the range compensator would be to use low cost 3-dimensional printer. This approach reduces manufacturing times with more efficient cuts and a more personalized process than a computerized milling machine.

The other issue of PAT plans necessarily requires a number of material components in the beamline, specifically scatters and collimators, proton interactions with these components result in the production of high-energy secondary neutrons. This is not just a PAT issue; in general, the conventional passive scattering systems have their limitations. To date, we have considered only the primary doses shown by the TPS. However, accurate comparisons of radiation-induced carcinogenesis among treatment modalities should also include calculations of secondary doses, such as the leakage dose in photon beam and the neutron dose in proton beam [21, 26]. Although proton therapy may result in reduced exposure of adjacent normal tissue to high and intermediate doses, the therapy may nonetheless result in an increase in low doses to the rest of the body (because of neutrons produced by the scattering components of passive scattering proton beams) which exceed those resulting from conventional photon treatment. Some technical issues, such as proton scattering from apertures, have been skipped as well [25]. In particular, most proton collimators are currently made out of brass or cerrobend which are high atomic mass material; a collimator made out of low mass material could significantly decrease the neutron dose and thus the cancer risks associated with secondary neutrons.

The clinical and practical use of PAT is still limited and technologically unique but clearly emerging as a desired and required mode for alternative treatment technique in proton beam. The PAT is accepted as important complementary methods to the conventional passive scattering and pencil scanning techniques. Of course the pencil beam scanning is a precise and efficient method as no field specific hardware is needed. The disadvantage of scanning is the higher sensitivity to organ motion compared to passive scattering [25]. The sensitivity to organ motion errors is the main reason why only well immobilized tumors- located in the head and neck, spinal cord and lower pelvis- have been treated using a scanning technique. Organ motion is therefore the single most important motivation to improve the utilization of scanning techniques. A major effort in the development of fast scanning techniques is needed to apply conformal beam scanning with a reasonably high number of target repainting. As a backup solution one should also develop simulated scattering. The approach to use a scanning system as the basic equipment could become in the near future practical steps, as scanning can create any dose distribution produced in a scattered beam [25]. The recent study also reported that the proton RBE exhibits a linear energy transfer (LET)-dependency and that this could play a role in treatment planning [27]. It suggested that proton-modulated arc therapy might allow simultaneous dose and LET painting of a target while delivering the dose in an efficient manner. We plan to examine the feasibility of performing proton-modulated arc therapy, choosing to compare simple passive-arc beam that clearly demonstrate the physical and biological properties of intensity-modulated arc beam used. This approach is possible that it provides a solution to the organ motion problem in pencil beam scanning.

## Conclusion

This study demonstrates that there is a potential role for PAT as an alternative technique to conventional proton therapy. PAT is not yet clinically available, but it will be more efficient and safer treatment in the future with additional research and development for commercialization.
